# Future schistosome hybridizations: Will all *Schistosoma haematobium* hybrids please stand-up!

**DOI:** 10.1371/journal.pntd.0008201

**Published:** 2020-07-02

**Authors:** J. Russell Stothard, Sekeleghe A. Kayuni, Mohammad H. Al-Harbi, Janelisa Musaya, Bonnie L. Webster

**Affiliations:** 1 Department of Tropical Disease Biology, Centre for Neglected Tropical Diseases, Liverpool School of Tropical Medicine, Liverpool, United Kingdom; 2 MASM Medi Clinics Limited, Medical Society of Malawi (MASM), Blantyre, Malawi; 3 Ministry of Health, Qassim, Kingdom of Saudi Arabia; 4 Department of Pathology, College of Medicine, University of Malawi, Blantyre, Malawi; 5 Malawi-Liverpool-Wellcome Trust Clinical Research Programme, Queen Elizabeth Central Hospital College of Medicine, Blantyre, Malawi; 6 Parasites and Vectors Division, Life Sciences Department, Natural History Museum, London, United Kingdom; University of Pennsylvania, UNITED STATES

Interrogating the genetic make-up of schistosome larvae (i.e., eggs, miracidia, and cercariae) originating from definitive or intermediate snail hosts with molecular DNA methods has, by noting unexpected interspecies hybrids, started a revolution in our appraisal of African schistosomiasis [1–4]. Here, two dominant species of human schistosome exist, *Schistosoma haematobium* and *S*. *mansoni*, which are transmitted by specific intermediate freshwater snails, *Bulinus* spp. for the former and *Biomphalaria* spp. for the latter. The two schistosomes cause either urogenital or intestinal schistosomiasis, respectively [5], and depending on local snail distributions, schistosome transmission zones in the aquatic habitat may or may not overlap [6]. Within the *S*. *haematobium* group, a further eight sister species are described with *S*. *intercalatum* and *S*. *guineensis* of medical importance, causing intestinal schistosomiasis, while others, such as *S*. *bovis*, *S*. *curassoni*, and *S*. *mattheei* occur in livestock, with the remaining species infecting wildlife. *S*. *mattheei* is also of medical interest for occasional infection and associated disease [7]. In contrast, *S*. *mansoni* has a single sister species, *S*. *rodhaini*, typically found in small rodents which can hybridize with *S*. *mansoni*, if given sufficient opportunity [2].

Across Africa, whilst the continental burden of coinfection with urogenital and intestinal schistosomiasis is not formally reported, at the local level, it often is [5, 8]. Inside a coinfected definitive host, an enigmatic but dynamic set of plausible worm-pairings can take place; these include a combination of both homo-specific (e.g., *S*. *haematobium* [♂] and *S*. *haematobium* [♀]) and various hetero-specific (e.g., *S*. *mansoni* [♂] and *S*. *haematobium* [♀], etc.) worm couplings [9]. Cross-specific couplings depend on worm competition, mating preference, and anatomical location(s) in the vasculature surrounding hepatoportal, urogenital, and intestinal systems [9]. As further hybrid variants come to light [2], some hetero-specific worm-pairing possibilities are of particular significance for currently known genetic introgression (e.g., *S*. *haematobium* and *S*. *intercalatum*) or more critically as yet unknown interactions (e.g., amongst various *S*. *haematobium*-hybrids themselves with or without *S*. *haematobium* or *S*. *mansoni* couplings). In contrast with experimental schistosomiasis [9], in which laboratory manipulation and direct dissection of adult worms can provide a sentry insight into interspecific pairings and hybridization potentials, our understanding of worm-pairing dynamics in the human host is entirely inferred by genotyping schistosome retrieved ova and/or miracidia [2]. This opens up biological conjecture on (incomplete) parthenogenesis, genetic introgression, and past and present hybridization processes [9]. Furthermore, our understanding of those schistosome pairings whose ova fall below current urine- or stool-detection thresholds is vague, as is our knowledge, by lack of schistosome-snail experimentation, of their (un)successful viability in aquatic habitats [10].

Control of schistosomiasis is of global importance and features in the World Health Organization (WHO) 2021–2030 neglected tropical disease (NTD) road map (see https://www.who.int/neglected_diseases/Ending-the-neglect-to-attain-the-SDGs--NTD-Roadmap.pdf?ua=1), with future disease-specific targets being set. Assumptions for successful control of *S*. *haematobium* include negligible capacity for viable genetic introgression(s) and maintenance of transmission in animal reservoirs. Seminal field-based parasitological surveys in Senegal, molecularly characterizing schistosome populations [1], have questioned these assumptions, bringing to light novel genetic interactions of *S*. *haematobium* and *S*. *bovis* [11]. With increasing geographical sampling, broader interspecies interactions with *S*. *curassoni* have been revealed [12], although a recent microsatellite analysis of Senegalese *S*. *haematobium* and *S*. *bovis* populations has shown no genetic admixing [13]. Nevertheless, the *S*. *haematobium*–*bovis* hybrid has been recently flagged in autochthonous transmission of urogenital schistosomiasis on Corsica [8]; neither inspected livestock nor rodents appear to sustain transmission locally, which is most likely of human-origin(s) and imported input(s) alone [14].

**Fig 1:**
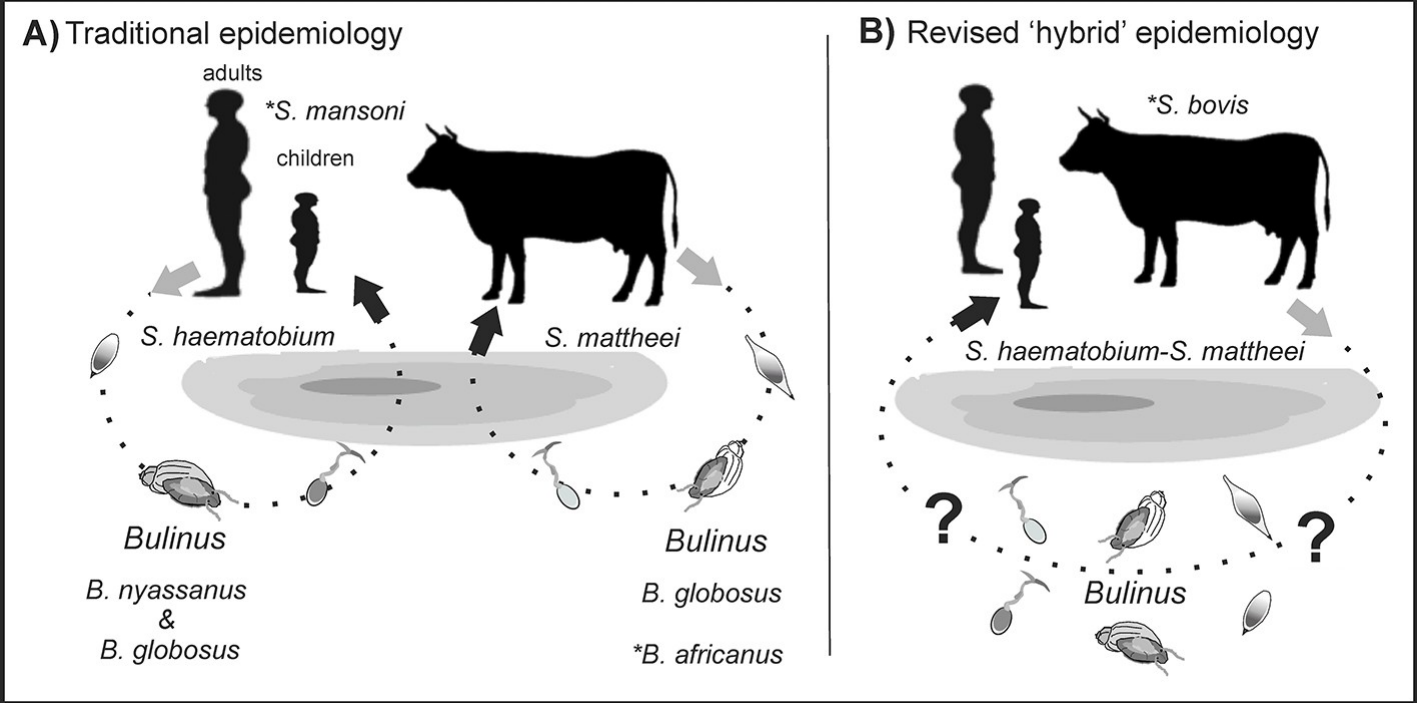
Reconciling schistosome hybridization with epidemiological models of schistosomiasis transmission. **(A)** The current model of urogenital schistosomiasis in Malawi involves discrete transmission cycles and does not formally take into account the importance of multi-species coinfections. **(B)** A revised model of urogenital schistosomiasis with overlapping transmission potentials with schistosome worms also being influenced or interacting with other species present (e.g., *S*. *mansoni* within coinfected people or *S*. *bovis* in cattle). Note *S. *mansoni*–intestinal schistosomiasis is now emerging along the Lake Malawi shoreline and is transmitted by *Biomphalaria* (which is not depicted here for brevity), *B. africanus has now been detected in the lake (MH Al-Harbi *personal observation*) and is a known intermediate host of several other *S*. *haematobium* group species, which might drive novel environmental transmission opportunities.

Interspecies hybrids are now being described in Malawi, Central Africa, where both *S*. *haematobium*–*bovis* and *S*. *haematobium*–*mattheei* combinations have been identified from ova retrieved from infected children [15]. This situation again challenges our current model of transmission, raising questions on how these hybrids first appeared and their current epidemiological infection cycles (see Fig 1). For example, the current transmission model of *S*. *haematobium* has discrete and nonoverlapping cycles with livestock schistosomes and also overlooks the importance of urogenital and intestinal schistosomiasis coinfection (Fig 1A). The presence of *S*. *mattheei* and *S*. *bovis* is currently inferred from its hybrid forms, as excreted from people, for there is neither formal reporting nor ad hoc inspections of bovine schistosomiasis. A revised transmission model of *S*. *haematobium* is therefore needed (see Fig 1B) with putative zoonotic transmission facilitated perhaps by the novel presence of other compatible snail species now being shown to occur in Lake Malawi.

Even though phenotypic suggestion of *S*. *haematobium*–*mattheei* hybrids (i.e., by egg morphology) was first reported over 70 years ago [16], being later confirmed biochemically and genetically in the laboratory [17, 18], it was then judged rare and not against a background of coinfection with *S*. *mansoni*. The latter species could be a cryptic infection driver altering worm behaviours by promoting mate exchange, for here in Malawi, intestinal schistosomiasis is emerging and appears very recent, being first noticed two years ago, driven by first colonisation of *Bi*. *pfeifferi* within the lake [19]. With these new aspects, novel epidemiological opportunities arise for multi-species coinfections (i.e., *S*. *haematobium*, *S*. *haematobium*–*mattheei* and *bovis*, and *S*. *mansoni*), driving plausible schistosome interactions, as meshed with ongoing environmental change. This includes regular or sporadic natural or manmade events such as cyclonic flooding, human migration, over-fishing, and increased pisciculture, where there have been significant, albeit largely unexplained, saltatory changes in snail fauna and schistosome-snail host ranges [19]. This clearly calls for further genetic scrutiny of schistosome infections in all definitive hosts locally, especially where multi-species coinfections are suspected as well as stepped-up environmental surveillance of infections in local snails for expanded schistosome-snail host ranges.

From our targeted epidemiological surveys of Malawian school children, an initial genetic analysis of a selection of schistosome eggs revealed an allopatric, geographically separated, distribution of *S*. *haematobium*–*mattheei* and *S*. *haematobium*–*bovis* hybrids although each hybrid was sympatric, geographically synonymous, with *S*. *haematobium* and *S*. *mansoni* [15]. Egg-patent prevalence of urogenital schistosomiasis was 25.6% (95% CI 16.7–34.4), some 10% of infected children shed atypical (i.e., morphologically unusual) *S*. *haematobium* eggs in urine. Egg-patent *S*. *mansoni* was also noted in several children’s stools as well as ectopic excretion in children’s urine, with the general prevalence of intestinal schistosomiasis increasing (more than 50% by urine-CCA testing), enabling coinfections to surge [15, 19]. Inspection of other demographic groups upon general surveillance of male genital schistosomiasis in fishermen, a urine egg-patent prevalence of *S*. *haematobium* (with typical egg morphology) was 17.1% (95% CI 4.8–22.2) although atypical *S*. *haematobium* eggs or *S*. *mansoni* eggs in urine were not found [20]. Upon real-time PCR analysis of semen with a genus-specific *Schistosoma* DNA probe, infection prevalence was much higher at 26.5% (95% CI 18.4–34.6), and, upon using urine-CCA tests, prevalence of intestinal schistosomiasis was 3.8% (95% CI 3.1–4.5) [19], indicative of coinfection in other community members. A targeted collection of stool from four fishermen led to the discovery of an atypical terminal-spined egg (*S*. *mattheei*?) which has not yet been subjected to molecular analyses, although a *S*. *mattheei* infection has been detected locally in *B*. *globosus* by molecular methods [20].

Clearly, multispecies schistosome coinfections are occurring here in Malawi with an underlying set of putative homo- and hetero-specific worm-pair coupling that enable new opportunities for known, but more importantly, as yet unknown, genetic interactions. The presence of the *S*. *haematobium*–*bovis* hybrid is particularly intriguing as the literature notes an absence of *S*. *bovis* greater than 10^o^ south of the equator. This demonstrates a more itinerant nature of this hybrid form outside the known range of *S*. *bovis* [8]. Since movement of infected livestock over such large distances is doubtful, more reasonable, like in the Corsica setting, is the introduction(s) of this hybrid from infected human migrants with autochthonous transmission in local snails which is, as of yet, to be formally observed. Better surveillance of schistosomiasis in local snails and livestock, particularly in cattle, is therefore needed.

Whilst genomic analysis of this *S*. *haematobium*–*bovis* hybrid is yet to take place, analysis of West African *S*. *haematobium*–*bovis* hybrids reveals several large 100 kb identical chromosomal regions, which is indicative of a single or very limited number of hybridization events given putative multiple rounds of subsequent meiosis [21]. An ancestral isolated genetic introgression event may not hold true for these Central African hybrid forms. Rather, we speculate it is contemporary, ongoing, and expanding with growing opportunities for interspecific crossings arising in coinfected people and associated livestock; thereby generating new genetic diversity over and above what is currently known regionally for *S*. *haematobium*. In terms of the epidemiology of *S*. *haematobium*–*bovis*, in Senegal it was noted that the frequency of hybrids, by village, was not associated with the prevalence of urogenital schistosomiasis but oddly was with the prevalence of intestinal schistosomiasis [11]. Referencing the Malawian *S*. *haematobium*–*mattheei* hybrid, we surmise that with increasing epidemiological opportunities for multi-species coinfections, both pre- (e.g., geography, host specificities, and anatomical sites) and post- (e.g., genetic factors and altered snail compatibility) zygotic species barriers are breaking down, allowing perhaps even broader schistosome species barriers to become eroded.

As the reproductive biology of the schistosome is unusual [4, 9], mention of JBS Haldane’s rule is worthy here as a biological framework to help decipher the directionality of hybridization (i.e., its mating asymmetry), for if only one sex of a species hybrid is inviable or sterile that sex is more likely to be the heterogametic one. By contrast to other trematodes, the schistosome is dioecious, not hermaphrodite, with the female, not the male, being the heterogametic sex (ZW[♀] and ZZ[♂]) [9]. It is also schistosome eggs, not the worms that stimulate inflammatory and fibrotic disease [5], hence any altered traits in eggs, and their anatomical sites of deposition, as well as in miracidia, could be important in terms of disease and environmental transmission. Notably, at least in the laboratory, viable offspring arise from all *S*. *haematobium* and *S*. *mattheei* combinations so far attempted, but this phenomenon did not spark increased public health vigilance even though heterosis in worm fecundity (i.e., increased egg-laying) was observed [9]. The newly reported *S*. *haematobium* group hybrids and assorted coinfection permutations in Malawi is now of fresh concern by enhancing future hybridization and disease potentials. For example, upon various plausible schistosome pairings such as *S*. *haematobium*–*mattheei* (♂) and *S*. *mansoni* (♀) or any back crossing of viable variants thereof could these worms with more eroded species-specific barriers now introgress? Of particular note, from experimental schistosomiasis is that viable progeny from *S*. *mansoni* (♂) and *S*. *mattheei* (♀) pairings has already been reported but were then thought, although not proven, to be parthenogenetic matriclonal lineages [9]. If not, could such introgressed progeny that arise in nature with altered disease then disperse, akin to *S*. *haematobium*–*bovis* migration, and subsequently intermingle with other populations? We should now be especially mindful of more distant species variants, particularly given ancestral genomic signatures [22] as well as more obvious recent ones, have been brought to light by detection of *S*. *mansoni*–*haematobium* hybrids in a migrant boy, even though the viability of their progeny was not determined [23]. Of recent note also is the report of an infection cluster of prepatent *S*. *haematobium*–*mattheei* hybrids, with evidence of *S*. *mansoni* infection, as detected in returned Belgium travellers from South Africa that were subjected to a more in-depth diagnostic investigation with molecular methods than usual routine practice [24].

It is an unfortunate situation that where schistosomiasis transmission occurs, there is typically only rudimentary parasitological surveillance and formal epidemiological inspection of animals is very rare [5, 25]. This is largely due to bottlenecks in adopting a “OneHealth approach.” Whilst atypical eggs, unusual in morphology, can alert, a precise inference of hybrids upon egg shape is fallible. Moreover, upon comparison with molecular methods, it grossly underestimates the true prevalence and the exact type of hybrids [26]. Nonetheless, our abilities to separate and identify unusual schistosomes, albeit adult worms or various larvae, with current molecular markers is also imperfect. Development of low-cost species and hybrid-specific DNA screening assays is now important to enable more rapid detection of hybrid schistosomes or the detection of genetic signatures of nonhuman schistosomes within clinical samples [26], particularly in relation to any changes in pathology or treatment efficacy. Current molecular identification and separation methods typically analyze eggs, miracidia, or cercariae, individually and, despite being extremely insightful, are often prohibitively expensive and very labour intensive and inspect only a few genetic loci at a time [2]. On the other hand, population genomics is the key to understanding hybridization events in time and space, with species-level studies essential to answer key questions in relation to public health (e.g., related pathology, transmission dynamics, drug efficacy, host specificity, and potential zoonoses) and to better understand schistosome evolution, hybridization, introgression, and reproductive biology.

**Fig 2:**
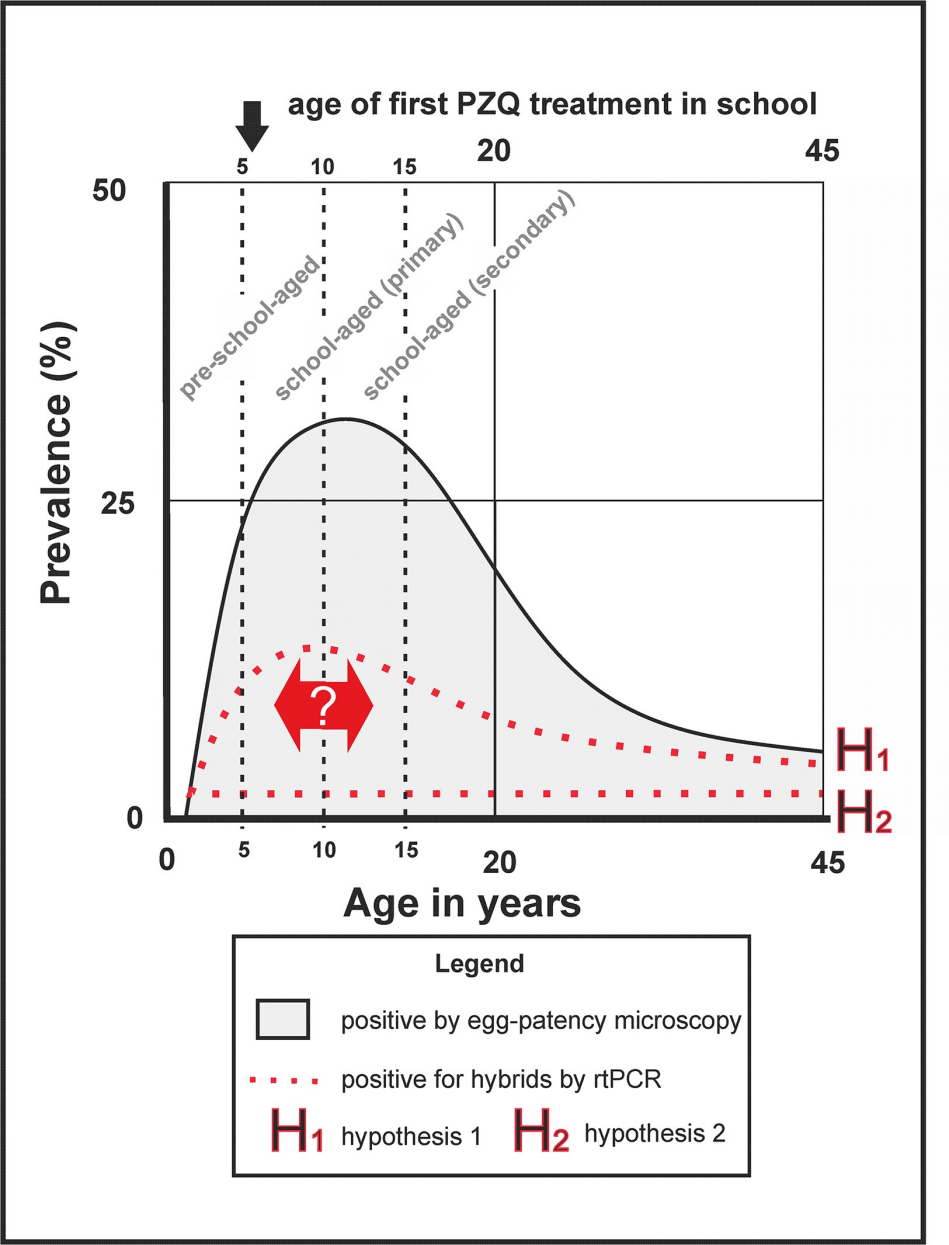
Highlighting knowledge gaps in the epidemiology and distribution of hybrid coinfections across a “typical’ community as current age-prevalence profiles are not known. This hypothetical scenario raises important epidemiological questions in the short- and long-term infection dynamics and needs for any future targeting of preventive chemotherapy control to any specific demographic groups (e.g., preschool-aged children) where hybrids may be, for example, more common. PZQ treatment campaigns typically commence in primary school, where the youngest children to receive treatment are usually older than five years of age. Thereafter, provision of PZQ treatment then follows either annual or biennial administration cycles. PZQ, praziquantel.

To highlight, in outline, the real epidemiological implications of these knowledge gaps in the detection and transmission of hybrids, an epidemiological postulate is provided in Fig 2, as based upon available survey information from Malawi [15, 19, 20]. Our inability to identify the presence and then tally the intensity of hybrid schistosome infections across a community severely hampers our appraisal of their epidemiological importance [10]. For example, we are currently unable to quantify if certain demographical groups are at more risk of hybrid infection and disease than others; or do the same or different demographic groups facilitate hybrid transmission or act as refugia; and most importantly, are hybrid parasites, as being boosted by zoonotic inputs, more resilient to preventive chemotherapy (PC), for example, by having altered life-history traits permitting more successful environmental transmission or are more tolerant to current praziquantel treatment regimens? Urogenital schistosomiasis, as detected by terminal-spined schistosome eggs in urine, typically commences in early childhood then rises to peak prevalence in teenage years, thereafter urine egg-patent infection wanes in older ages [25] as within host worm burdens decline or as worm-pair fecundities reduce. This “classic” curve gives rise to a well-known age-profile peak resultant from a variety of dynamic and interacting factors e.g., water contact, partial immunity, host and/or schistosome mortality, etc. [25]. We presently do not know if there is, or is not, a “classic” age-prevalence profile for hybrid coinfections, i.e., occurrence of a hybrid nested within a patent *S*. *haematobium* and/or *S*. *mansoni* infection. However, we can envisage two likely scenarios as outlined by the dashed red lines: H1—the age-profile of hybrid coinfection follows that of a “classic” monospecific infection but is reduced in amplitude in line with diminishing ova-shedding, an accepted feature of schistosomiasis—or H2—the age-profile is constant (or sporadic) across ages and does not track a “classic” monospecific infection, perhaps by being a less facultative transmission process in local snails. In **H1,** there is also the possibility of an epidemiological “peak-shift” where the age-profile maximum prevalence is either advanced or retarded in reference to the monospecific infection (red arrow). If so, there could be many reasons for this; for example, hybrid worms may have a faster environmental transmission dynamic (i.e., a left-shift) or hybrid worms live longer (i.e., a right shift). A similar age-prevalence argument based upon “peak-shift” [25] highlights a parallel knowledge gap that is pertinent to a OneHealth perspective for the age-prevalence burden of schistosomiasis in livestock is unknown. For example, although Savassi et al. [27] recently demonstrated domestic cattle as natural hosts of *S*. *haematobium* and *S*. *haematobium*-*S*. *bovis* in Benin, they did not comment on the age of their cattle examined but they did observe altered shedding times of schistosomes from infected snails, noting their importance as environmental transmission drivers.

To close, for a more thorough surveillance of hybrid schistosomes in Africa, we strongly advocate a OneHealth approach. Where possible, this could include noninvasive larval sampling of schistosomes from livestock with spatial tracking of sentinel animals within mobile infected herds for real-time monitoring of infection dynamics, with better snail surveillance at well-noted animal water contact sites. Upon postmortem inspection of animals at slaughter or upon selected cull, we should also be on guard for any ectopic worms in the vasculature of either urogenital or intestinal systems as well as how ova-contaminated water from carcasses might cycle back into aquatic habitats from abattoir effluents. Without doubt, the search for emergence of further schistosome hybrid combinations in humans, alongside our growing ability to detect them, is increasing; to help us in this quest, simply put, can all *S*. *haematobium* hybrids please stand-up!
